# The Synovial Membranes in Pyæmia

**Published:** 1872-09

**Authors:** Robert Hamilton

**Affiliations:** Liverpool


### The Synovial Membranes in Pyæmia.

By Robert Hamilton, F. R. C. S., Liverpool.

"After a brief account of the views at present held by the majority of writers on pyæmia, as to the origin of the morbid changes found in this disease, the author dwelt upon the fact of the synovial membranes being generally attacked in pyæmia. This circumstance had not, hitherto, attracted sufficient attention, though it was extremely probable that the first step in those forms of surgical pyæmia commonly met with in hospital practice, was to be found there. All the cases occurring at the Liverpool Southern Hospital, for the last thirteen years, of which particulars had been kept, had some joint-affection. In some, the pathological changes in the joint were slight, in others most extensive. That the disease observable in the joints began in the synovial membrane, was rendered probable from the character of the pain, and from the appearances found after death.

The poison of pyæmia, as observed in hospital practice, was *sui generis.* Whether generated in the system from a combination of constitutional and surrounding conditions, or entering as a specific germ through a wound, it had a special affinity for certain structures, and to these it passed at once. The structures were assumed to be the synovial membranes. In what this affinity or attractiveness consisted we were as yet ignorant, and could but illustrate it by what took place in other diseases, such as the cholera poison, and the scarlatinal poison, or the producer of tetanus; whether it is a something evolved within the system, or introduced from without, it went at once to certain nerve-tissues, and in them created changes which set up other morbid action. Many drugs illustrated the same fact. Strychnia, when swallowed, or when subcutaneously injected, had but one form of action—it affected nerve-tissue only.

The strong analogy between pyæmia at the commencement, and acute rheumatism, had often been observed. In both there were the rigors, the fever, the rapid pulse, the profuse sweating; but, above all, there were the pain and swelling of one or more joints. it seemed probable that, in both, an entity had entered the system, whose habitat is the joints. It was not known in what consisted the difference between the two poisons, so that the one, as a rule, eventuated in recovery, and the other in a train of pathological changes whose termination was death.

In both cases the tissue first affected was the synovial membrane. The abnormal action induced in it led to an increased secretion of synovia, probably unaltered in its character and constituents in rheumatism, but abnormal in pyæmia.

In the case of the synovial fluids there was, in most joints, a limit to its quantity; so tightly was the synovial sac compressed by the yielding tissues, that an amount of tension quickly ensued, which led to a forced absorption of some of the effused fluid, and then ensued in acute rheumatism, and probably, as a necessary sequence, an extension of the disease to other synovial sacs, and often to the pericardium, a serous membrane, but closely allied, in its nature, to a synovial membrane.

This augmented synovial fluid, in rhenmatism, was bland an innocuous—a mere increase of the natural secretion; but, in pyæmia it was in a decomposing state, developing rapidly germs of a lower organization, and when such a fluid had been absorbed, and in its course reached the minute capillaries of the lungs, some of its morbid cells coagulated the fibrine of the blood there, and became arrested, and thus were formed the nuclei with which the lungs were studded, around which more fibrine was deposited, and the pathological changes followed, as described by Virchow and others.

In conclusion, the author stated that, in limiting the paper to a consideration of the fact of the synovial membianes being the tissues first affected in many forms of pyæmia, he had not lost sight of the probability of many other phases of pyæmia having their beginnings in one or other of the serous membranes, these two membranes being closely allied in their microscopic characters. —*London Medical Press and Circular*, June 5th, 1872." (*Nashville Journal of Medicine and Surgery.*)

